# Supplementary material for: Non-Palpable Breast Cancer: A Targeting Challenge–Comparison of Radio-Guided vs. Wire-Guided Localization Techniques
Source: Biomedicines. 2024 Oct 27;12(11):2466. doi: 10.3390/biomedicines12112466 (PMC11592315; doi:10.3390/biomedicines12112466)
Supplement: Supplementary file 1 [file biomedicines-12-02466-s001.zip › biomedicines-3270276-supplementary.pdf]

Statistical Results

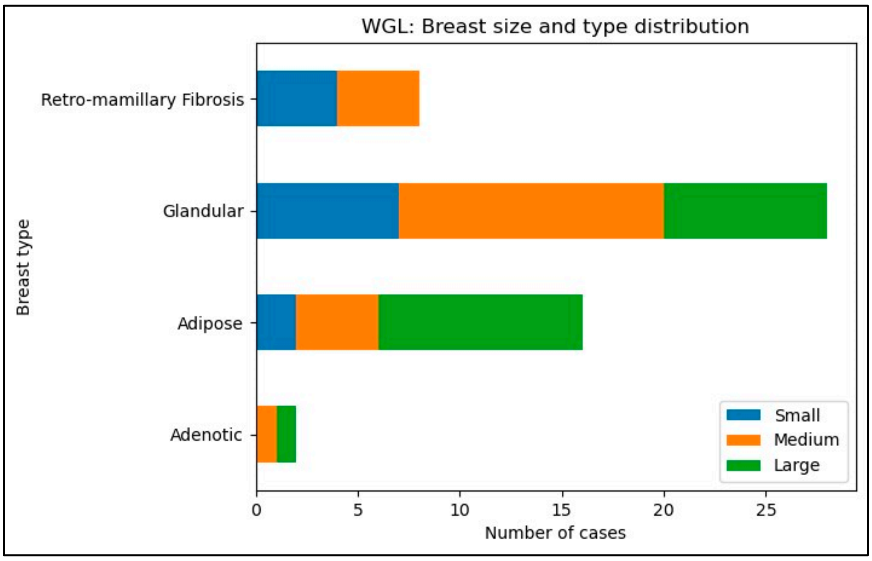

Figure S1. Breast size and type distribution in the WGL group.

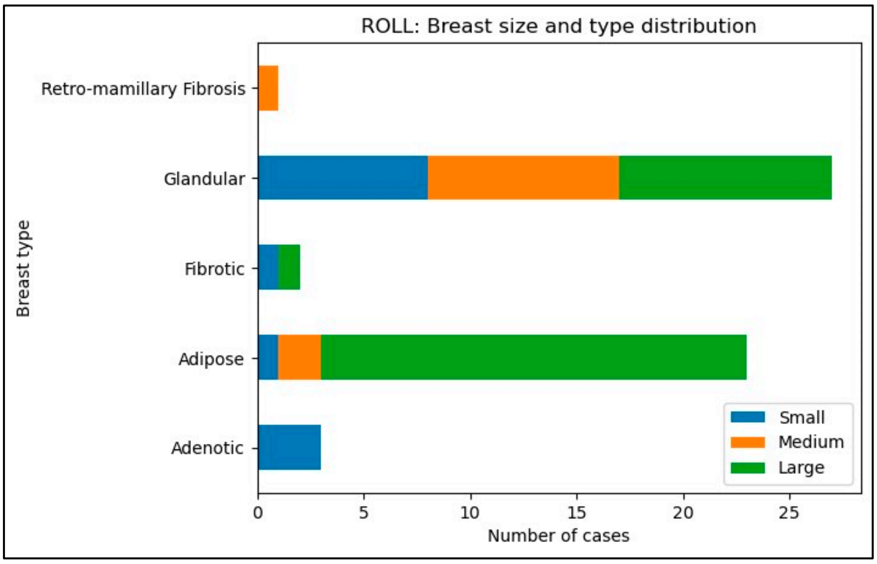

Figure S2. Breast size and type distribution in the ROLL group.

1. Age of patients in the two groups examined

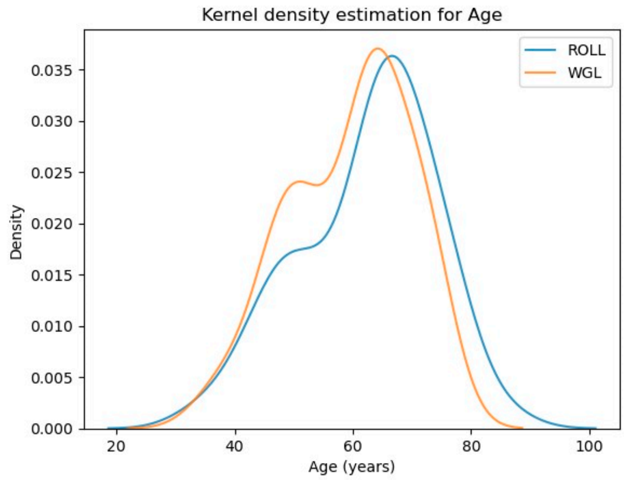

Figure S3. Ages of patients in the examined groups at the time of the operation. The mean age of the patients was 62.4 ± 11.31 years in the ROLL group and 59.56 ± 10.1 years in the WGL group, with 95% confidence intervals of (59.37, 65.43) and (56.8, 62.32), respectively.

2. BMI of patients in the two groups examined

The mean BMI was 25.5 ± 4.2 in the ROLL group and 26.5 ± 4.1 in the WGL group (*p*-value: 0.5932).

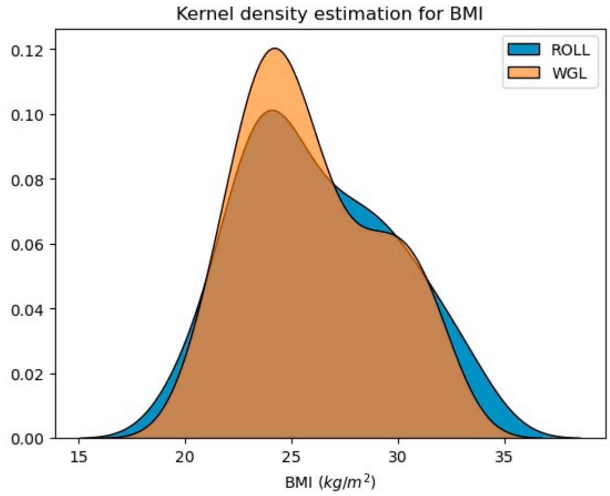

Figure S4. BMI of patients at the time of the operation. The mean BMI was 26.24 ± 3.61 kg/m2 in the ROLL group and 25.89 ± 3.19 kg/m2 in the WGL group, with 95% confidence intervals of (25.27, 27.2) and (25.01, 26.76), respectively. There were no significant differences in age or BMI distributions between the groups.

3. Preoperative difficulty of marking non-palpable breast lesions in each procedure—radiologists' perspective

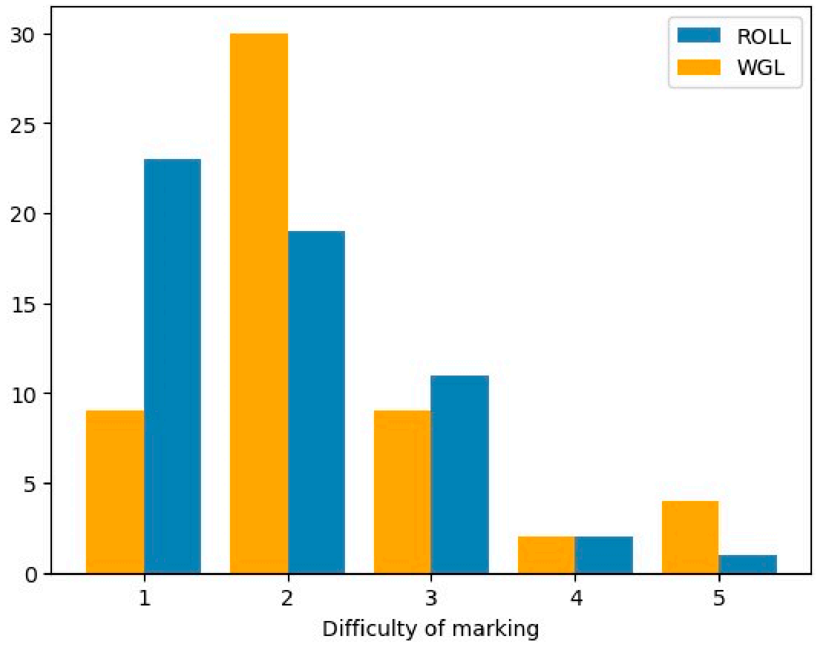

Figure S5. Preoperative difficulty of marking non-palpable breast lesions. ROLL: min: 1, mean: 1.75, median: 1.5, max: 5, standard deviation: 0.96. WGL: min: 1, mean: 2.31, median: 2, max: 5, standard deviation: 0.97. The preoperative difficulty of lesion marking in case of ROLL is significantly lower than in the case of WGL (Mann–Whitney, *p*-value: 0.0002).

4. Duration of marking procedures

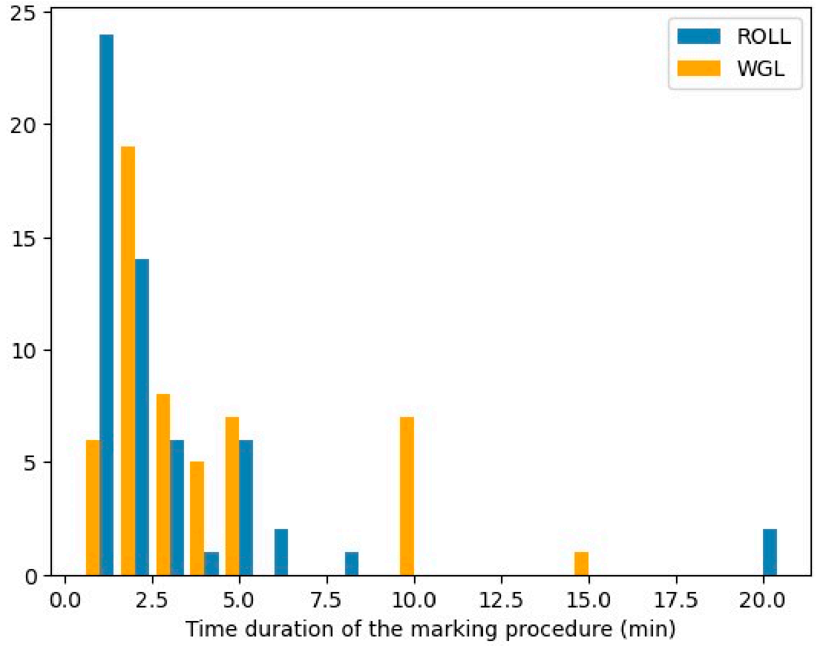

Figure S6. Duration of marking the non-palpable tumors. ROLL: mean: 2.93 min, standard deviation: 3.71. WGL: mean: 3.92 min, standard deviation: 3.16. The duration of localization in case of ROLL is significantly lower than in the case of WGL (Mann–Whitney, *p*-value: 0.001).

5. The pain of tumor marking with each technique—Patients' perspective

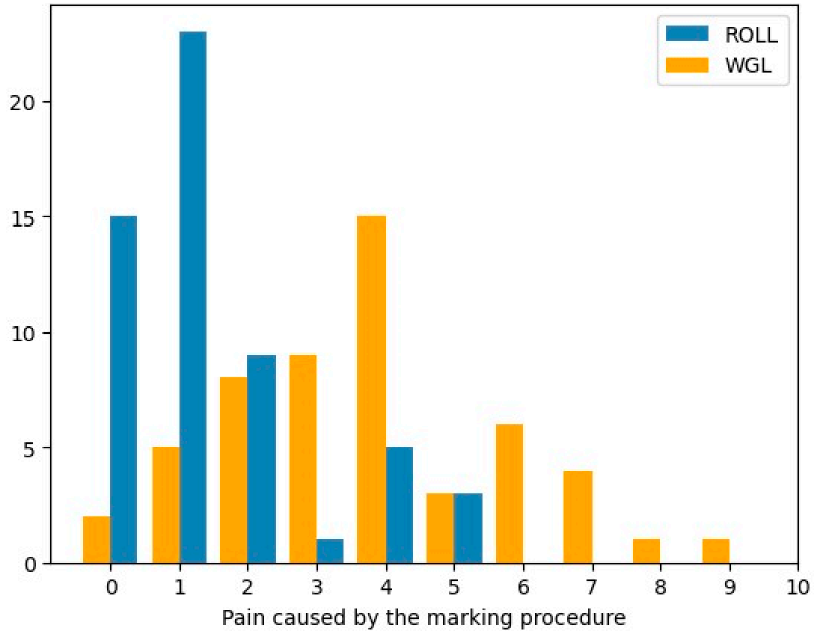

Figure S7. Pain of the tumor marking procedures.

The pain of tumor marking with each technique—Patients' perspective

Pain was rated by the patients themselves using the Wong-Baker scale [1] from 0 (no pain = no hurt) to 10 (maximum pain = hurts worst).

ROLL: min.: 0, mean: 1.41, median: 1, max: 5, standard deviation: 1.42

WGL: min: 0, mean: 3.78, median: 4, max: 9, standard deviation: 2.03

Pain during ROLL marking is significantly less than during WGL (Mann–Whitney test p-value: 0.0000).

6. Operative difficulty of lesion localization—surgeons' perspective

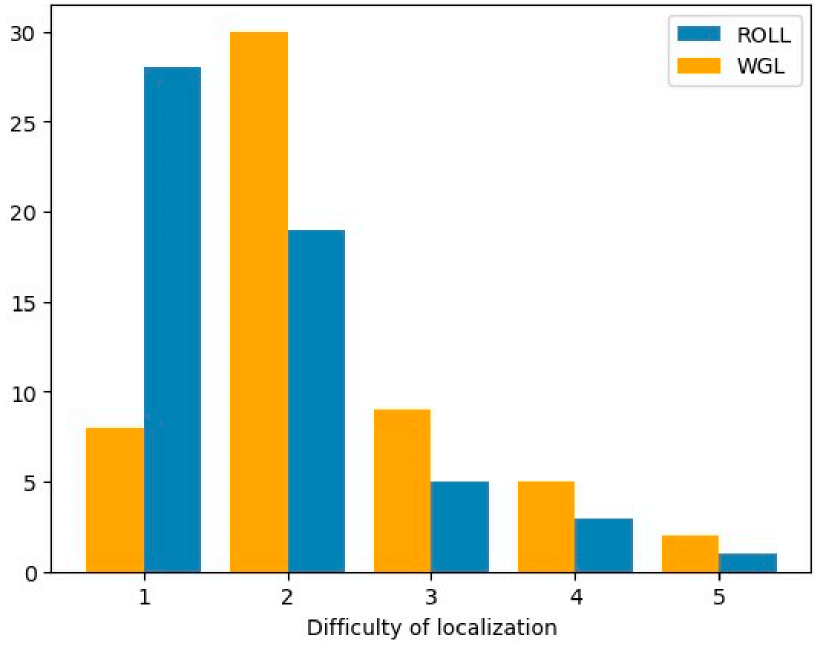

Figure S8. Operative difficulty of lesion localization. ROLL: min: 1, mean: 1.91, median: 2, max: 5, standard deviation: 0.96. WGL: min 1, mean: 2.29, median: 2, max: 5, standard deviation: 1.04. The

operative difficulty of lesion localization in case of ROLL is significantly lower than in the case of WGL (Mann–Whitney, *p*-value: 0.0197).

7. Time (duration) of operation (min)

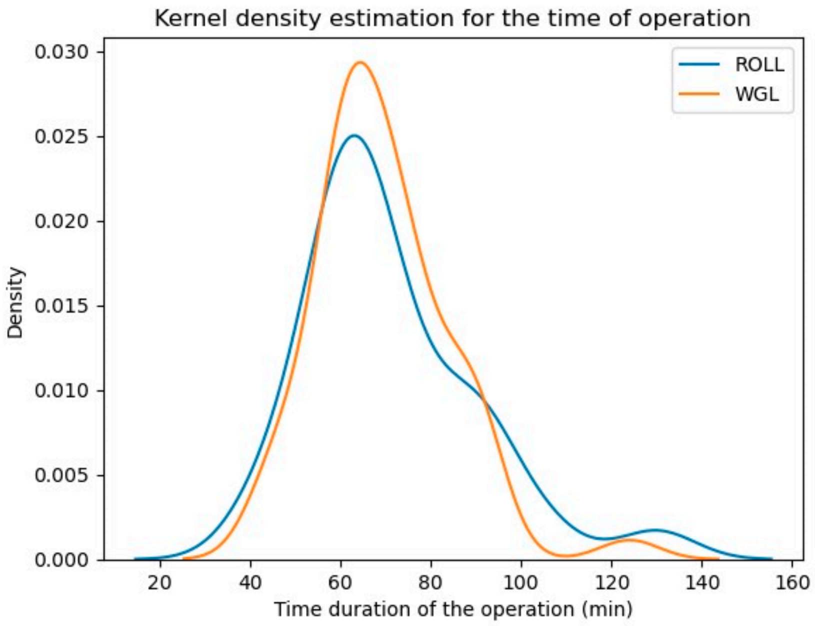

Figure S9. Duration of the operations in each group. ROLL: mean: 71.05 min, standard deviation: 18.92. WGL: mean: 69.26 min, standard deviation: 14.51. The 95% confidence intervals for the mean time are (65.99, 76.12) and (65.3, 73.22), respectively.

There is no significant difference between the distributions of the duration of operation (*p*-value: 0.9593), there is no significant difference between the mean time (according to the Kolmogorov–Smirnov test and two-sample t-test, Welch's version, *p*-value: 0.5771).

8. Weight of the specimen

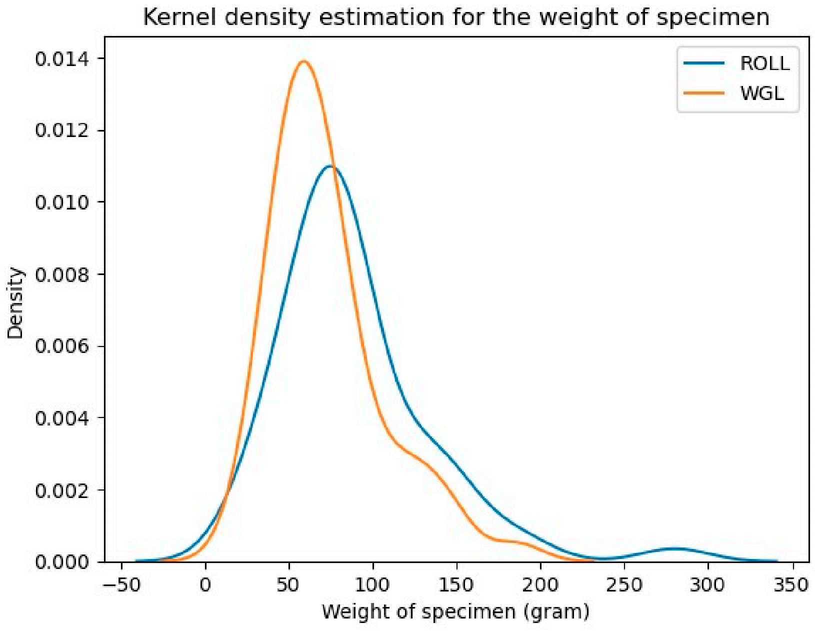

Figure S10. Weight of specimens in the two groups. ROLL: mean: 88.48 g, standard deviation: 45.25. WGL: mean: 72.43 g, standard deviation: 33.55. The 95% confidence intervals for the mean weight are (76.36, 100.6) and (63.27, 81.58), respectively.

The volume of the removed tissue, represented by its weight, was not different in the two groups, based on the Kolmogorov–Smirnov test. However, while there is no significant difference between the weight distributions (p-value: 0.0637), this p-value is quite close to the margin (0.05), and the Kolmogorov–Smirnov test is conservative. Thus, we applied other tests: based on the Cramer–von Mises test (p-value: 0.0162) or on the Mann–Whitney test (p-value: 0.019), there is a significant difference between the distributions, which means that the average specimen weight was greater in the ROLL group. In addition, the mean value of WGL is significantly smaller (p-value: 0.0183).

For clarity, we provide a summary of the statistical comparisons presented in Tables 5 and 6. 'No difference' indicates no statistically significant difference at the 5% level, while 'better' indicates a lower failure rate. The corresponding p-values are also provided.

Table S1. Comparison of SLN marking failure.

Localization	Isotope Marking Failure	Patent Blue Marking Failure
Lower-inner q.	WGL better (0.0265)	no difference (0.0847)
Upper-inner q.	no difference (0.0607)	WGL better (0.0017)
Central	-	-
Lower-outer q.	WGL better (0.0451)	no difference (0.1812)
Upper-outer q.	WGL better (0.0155)	no difference (0.1169)

For clear visualization of the situation, we constructed confidence intervals for the failure rates (using Wald estimation). Graphically, if the mean (midpoint of the interval) is included in the other interval, then there is no significant difference at the 5% level. Note that Wald estimation produces narrower intervals than Student's t-test.

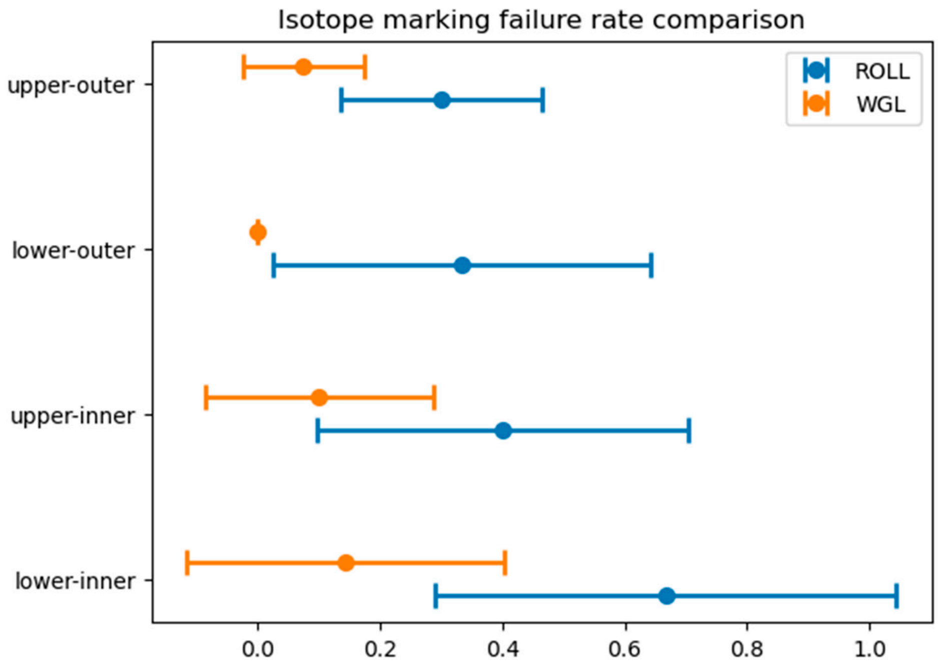

Figure S11. Confidence intervals for comparison of WGL and ROLL isotope marking failure rates.

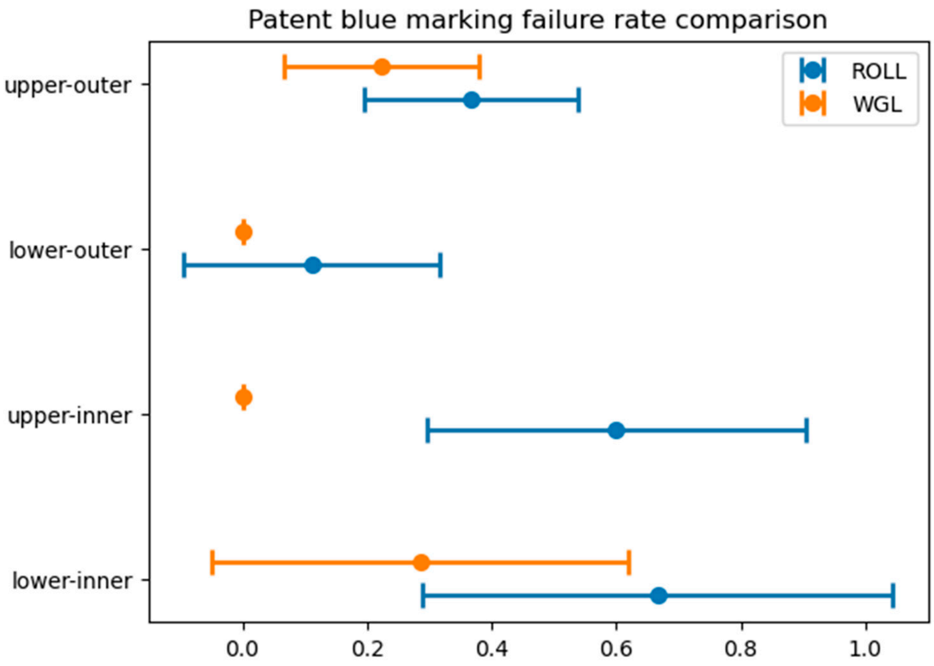

Figure S12. Confidence intervals for comparison of WGL and ROLL patent blue marking failure rates.
